# Acknowledgement to Reviewers of *Journal of Imaging* in 2019

**DOI:** 10.3390/jimaging6010003

**Published:** 2020-01-17

**Authors:** Journal of Imaging Editorial Office

**Affiliations:** MDPI, St. Alban-Anlage 66, 4052 Basel, Switzerland

The editorial team greatly appreciates the reviewers who have dedicated their considerable time and expertise to the journal’s rigorous editorial process over the past 12 months, regardless of whether the papers are finally published or not. In 2019, a total of 79 papers were published in the journal, with a median time to first decision of 21 days and a median time from submission to publication of 51 days. The editors would like to express their sincere gratitude to the following reviewers for their generous contribution in 2019:
Abdellatif, MohamedMarita, TiberiuAdler, AmirMartin, ArnaudAfonso, ManyaMartin, IainAhmad, MuhammadMartins, GabyAlex, AneeshMassa, AndreaAlfalou, AymanMassanet, SebastiaAliotta, FrancescoMatthews, LorinAllegra, DarioMayerich, DavidAlparone, LucianoMesselodi, StefanoAndreić, ŽeljkoMeyer-Baese, AnkeArines, JustoMilej, DanielAtaman, ÇağlarMiyata, TakamichiBae, Eui WonMolinara, MarioBará, SalvadorMondal, Suman BBarada, DaisukeMorrow, IvorBarentine, John C.Moseev, DmitryBarry, David JMutschler, ChristopherBeltran, CarlosNagata, FusaomiBernardes, RuiNayak, RohitBevacqua, Martina T.Netzel, HenrykaBialkowski, KonstantyNevin, AustinBlanco Heras, DoraNewson, AlasdairBlasinski, HenrykNguyen, BinhBoulgouris, Nikolaos V.Nguyen, ThanhBourgeois, SteveNguyen, Tuan T.Buch, Anders GlentNomura, TakanoriBurrascano, PietroNunes, PauloByun, HyeranNuutinen, MikkoCabaret, LaurentOikawa, KenichiCalvo-Zaragoza, JorgeOrtolani, SergioCarlson, Lars-AndersPagliarulo, VitoCarrara, FabioPanáková, DanielaCelona, LuigiPark, JaehwaChang, JiyulPavone, Santi ConcettoChen, Bo-HaoPedersoli, MarcoChen, ChenPepe, Francesco V.Chen, MinsiPerra, CristianChoi, HojongPerri, StefaniaChoi, Woo JunePiana, MicheleChuang, Yung-YuPirri, FioraCinzano, PierantonioPont, SylviaClement, YuenPopov, VladimirCoakley, Kevin J.Pradeep, BuddharajuCozot, RemiPrasath, SuryaCozzolino, DanielPresotto, LucaCrocco, LorenzoPutnam, AndrewDaffara, ClaudiaQi, GuanqiuDasygenis, MinasQueau, YvainDe Bernardi, ElisabettaRadonic, VasaDe Sousa, Jose T.Raffaeli, RobertoDeng, LeiRai, RobbaDi Massa, Giuseppe Ramchandani, DivyaDi Nunzio, LucaRandazzo, AndreaDiez, YagoRasti, PejmanDurou, Jean-DenisReiser, DavidEibert, ThomasRezazadegan Tavakoli, HamedEmberton, SimonRibas, Salvador J.Ezekiel, SoundararajanRodríguez, AiramFarhad, MerchantRohrbach, AnnaFarid, Muhammad ShahidRoig, BernardinoFernandes, CarlosRomeo, StefaniaFernández-Caballero, AntonioRoyle, StephenFerrández Vicente, José ManuelSaeed, KhalidFhager, AndreasSafonov, IliaFilip, JiriSalewski, MirkoFisher, RobertSalido, JesúsFontana, RaffaellaSanchez, AlejandroFrances-Villora, JoseSaur, GünterGällström, AndreasSavino, AlessandroGao, ShangceSawall, StefanGarrett, Joseph L.Schabowicz, KrzysztofGennaro, ClaudioSchiopu, IonutGiacomelli, MichaelSesma-Sara, MikelGomez-Donoso, FranciscoSharma, HarshitaGonzález-Hidalgo, ManuelShen, YaochunGoree, JohnShi, JieGraff, MarieSimon-Chane, CamilleGrammalidis, NikosSkalski, AndrzejGrana, CostantinoSolis Alfaro, JorgeGrosse, MircoSoltaninejad, MohammadrezaGuerrero, PaulSoria, XavierHadar, OferStevenson, GordonHanly, Joseph J.Strokina, NataliyaHatt, MathieuStrömberg, TomasHe, LifengTahmassebi, AmirhessamHeddleston, JohnTakahashi, TomohiroHelbig, MarkoTankam, PatriceHelbin, PiotrTarasov, AndreiHirayama, RyujiTen Bosch, L.F.M.Hoppe, AdamTheodorakeas, PanagiotisHung, Li-WeiThoma, MarkusHuynh-The, ThienTiberi, GianluigiImiya, AtsushiTicos, Cătălin M.Ipek, ÖzlemTimmins, ClaireItrić, KatarinaTobon, JorgeJaworska, TatianaTsiklauri, DavidJeffrey, IanTsinos, Christos G.Jørgensen, Jakob SauerTumurbaatar, TserennadmidJoslin, ChrisTurkington, AliceJuan, GuillermoUbaru, ShashankaJung, Ki-HyunUkwatta, ErangaKelly, ChristopherUpneja, RahulKeszöcze, OliverVallez, NoeliaKhatibi, SiamakVan Eijnatten, MaureenKhmaladze, Alexander T.Venkataraman, RajeshKiller, CarstenVozel, BenoitKim, KyungsangWang, KangKirchner, ThomasWang, QianKnapek, ChristinaWilliams, JeremiahKocifaj, MiroslavWoods, RogerKolláth, ZoltánYasukawa, ShinsukeKonda, PavanYeom, JiwoonKronreif, GernotYoon, JongheeKurrant, DougYu, HuiKyba, ChristopherYu, WeimiaoLe Pendu, MikaelYu, XinLee, GueesangZanetti, MassimoLee, Sang-HeonZeng, KaiLee, SoochahnZhang, HeyeLeeser, MiriamZhang, YuLeone, GiovanniZhong, YuLi, XiujunZhou, JunLima, Raul CerveiraZhurov, Alexei I.López-Quintero, Manuel IgnacioZilberti, LucaMainprize, JamesZolliker, PeterMandal, Subhamoy


